# Ulceration Endocarditis Consecutive to Gonorrhœa

**Published:** 1892-12

**Authors:** 


ULCERATIVE ENDOCARDITIS CONSECUTIVE TO GONORRHŒA.—His (*Berliner klin. Wochenschr.*, 1892, No. 40, p. 993) has reported the case of a man, nineteen years old, who, shortly after apparent recovery from an attack of gonorrhœa of but ordinary intensity, was seized with a chill, followed by an eruption of small, redish spots on various parts of the body, some of which were hemorrhagic. The heart became increased in size, and a loud, blowing systolic murmur, previously not detected, could be heard at the apex, but with greatest intensity in the course of the aorta. The temperature rose to 104.9 deg. The febrile movement was remittent The urine was excreted in increased quantity, but contained no abnormal ingredients. At no time was there pain in the perineum or testicles. The patient grew progressively worse and died from heart failure. At the post-mortem examination hemorrhages were found beneath the skin and the various mucous and serous membranes, and in the lungs, liver, testicles, cerebellum, and medulla oblongata, while the spleen, kidneys, and lungs contained infarcts. Thrombi were present in the public plexus of veins. The heart was enlarged, the left ventricle hypertrophied. Upon the right aortic semilunar leaflet were seated numerous excrescences, to which masses of fibrin and recent coagula were attached. Similar masses were seated upon the anterior half of the left leaflet, and a single nodule was present upon the posterior leaflet. Here and there were small areas of ulceration. The aorta presented evidences of inflammation. The other valves and orifices were normal. Microscopic examination disclosed the existence of an interstitial myocarditis, with puriform softening of a thrombus at the apex. Gonococci were looked for in the various lesions, but with doubtful results. In the tissues of the diseased aortic valve were found organisms that bore a close resemblance to gonococci.—*Am. Jour. of Med. Sciences.*

